# An interpretable machine learning-based approach: development, validation, and clinical utility for distant metastasis prediction in PTC

**DOI:** 10.3389/fendo.2026.1779102

**Published:** 2026-04-29

**Authors:** Ruijie Sun, Yuhui Ma, Yushan Jiang, Xiaoguang Li

**Affiliations:** 1 Department of Otolaryngology, Qilu Hospital of Shandong University (Qingdao)​, Qingdao, Shandong, China; 2 Department of Ultrasound, Qilu Hospital of Shandong University (Qingdao)​, Qingdao, Shandong, China; 3 Department of Ultrasound, Jimo People’s Hospital of Qingdao​, Qingdao, Shandong, China

**Keywords:** LightGBM algorithm, machine learning, metastasis, SHAP (shapley additive explanation), thyroid cancer

## Abstract

**Background​:**

Papillary thyroid carcinoma (PTC) constitutes 80–90% of all thyroid malignancies. Despite its generally favorable prognosis, 20–30% of PTC patients present intermediate/high-risk features, increasing distant metastasis risk. Traditional clinicopathological predictors (e.g., TNM stage, tumor size) have limited accuracy in forecasting metastasis, creating a need for more precise prediction tools.​.

**Methods​:**

A total of 2,452 PTC patients (diagnosed 2015–2023, follow-up ≥6 months) were enrolled, with data including clinical, pathological, laboratory, and ultrasound indices. Feature selection integrated LASSO, RFE, and ReliefF, identifying 7 core features. Nine machine learning (ML) algorithms were compared; SHAP analysis was used for interpretability. External validation included 432 patients, and a Django-based prediction website was developed.​.

**Results​:**

The LightGBM model exhibited optimal performance: test-set AUC = 0.886, accuracy = 0.887, and external validation AUC = 0.758. SHAP analysis identified extrathyroidal invasion (Mean |SHAP| = 0.1329) and thyroglobulin antibody (TgAb, Mean |SHAP| = 0.0981) as top predictors. Tumor size showed a nonlinear association with metastasis, and the model had a 93.6% negative predictive value (NPV) for excluding low-risk patients.​.

**Conclusions​:**

This interpretable ML model outperforms traditional predictors, effectively supporting clinical risk stratification and personalized treatment decision-making for PTC patients, with potential for broad clinical application.​.

## Background

### Disease status and epidemiology

Thyroid cancer represents the most common endocrine malignancy worldwide, with incidence rates increasing steadily in recent years (1). Papillary thyroid carcinoma (PTC) accounts for approximately 80–90% of all thyroid malignancies, making it the most prevalent histological type of thyroid cancer (1). Although PTC generally carries a favorable overall prognosis, approximately 20–30% of patients present with intermediate or high-risk features, placing them at substantially elevated risk for recurrence and distant metastasis (1). Distant metastasis, particularly to bone and lung, represents a major determinant of long-term patient survival and overall prognosis in PTC; prior studies have demonstrated that the occurrence of distant metastasis dramatically reduces the 5-year survival rate from over 95% to below 50%, substantially compromising quality of life and survival outcomes (2). Among patients with differentiated thyroid cancer (DTC), although the overall prognosis remains favorable, approximately 15–20% experience distant metastasis during follow-up (3).

Traditional clinicopathological features (such as TNM stage, extent of invasion, and tumor size) have demonstrated limited accuracy in predicting distant metastasis (1, 4). This limitation primarily stems from the fact that existing risk stratification systems are based on clinical experience rather than individualized data-driven analysis, and single clinical parameters cannot comprehensively reflect the complex biological behavior of tumors; furthermore, patients exhibit substantial heterogeneity in genetic background and immune status (4). Guo and colleagues demonstrated that age, tumor size, intrathyroidal dissemination, and central lymph node metastasis serve as independent predictive factors for lateral lymph node metastasis, suggesting the necessity of constructing a comprehensive multifactorial prediction model to enhance the precision of risk assessment (4). Therefore, there is an urgent clinical need to develop more accurate and individually tailored models for predicting distant metastasis in order to identify high-risk patients at initial diagnosis, thereby optimizing clinical decision-making and treatment strategies (1, 2, 4).

### Current related research and applications of machine learning

In recent years, machine learning has achieved significant advances in clinical applications (5, 6), particularly in predicting the prognosis of thyroid cancer, providing novel pathways for precision medicine. Huang and colleagues conducted research utilizing the Surveillance, Epidemiology, and End Results (SEER) database, enrolling over 10,000 patients with differentiated thyroid cancer, and developed a machine learning model for predicting bone and lung metastasis (7). This study compared multiple algorithms including random forest, gradient boosting, and XGBoost; the XGBoost model achieved an AUC of 0.988, substantially exceeding the logistic regression baseline of 0.756 (7). Key predictive variables included tumor size, radiation therapy, surgical intervention, histological type, TNM stage, laterality, race, and family income, findings that highlight the important role of socioeconomic factors in cancer prognosis (7).

Dai and colleagues conducted a multi-omics analysis integrating genomic, proteomic, immunological, and clinicopathological data, and applied eight different supervised machine learning models (including support vector machine, random forest, and logistic regression) to identify risk indicators for lymph node metastasis in stage N1b papillary thyroid microcarcinoma (PTMC) (8). This study identified the neutrophil-to-lymphocyte ratio (NLR) as the most important independent predictive factor (OR = 2.12, *p* < 0.01), with an AUC of 0.852, demonstrating strong discriminatory ability (8). Gu and colleagues further validated the significant association between immune-inflammatory markers (NLR and platelet-to-lymphocyte ratio [PLR]) and lymph node metastasis (9). Additionally, serum inflammatory markers such as high-sensitivity C-reactive protein (hs-CRP) and lymphocyte percentage demonstrated significant predictive value, suggesting close association between immune-inflammatory response and PTC metastasis; multivariate analysis by Wang and colleagues further confirmed these findings (9, 10).

Explainable artificial intelligence (XAI) and SHAP analysis have received increasing attention in medical AI in recent years (11). Feng and colleagues established a gradient boosting machine (GBM) model to predict lymph node metastasis in PTC and employed SHAP analysis to explore the model’s decision-making mechanisms in depth, identifying the lymphocyte-to-monocyte ratio (LMR) as the most important predictive factor with a mean SHAP value of 0.156 (11). The innovation of this study lies not only in predictive performance but also in emphasizing model interpretability, enabling clinicians to trust and accept the model in real clinical practice (11). Shen and colleagues developed an LLNM-Net deep learning multimodal model integrating ultrasound images, radiological reports, pathological findings, and demographic data, achieving an AUC of 0.944 in multicenter testing, surpassing the 64.3% accuracy rate of expert human practitioners (12). This study revealed that tumors located less than 0.25 cm from the thyroid capsule carry a >72% risk of metastasis, highlighting the importance of tumor spatial location; Martinez and colleagues validated similar findings in radiomics-based deep learning research (12, 13).

Li and colleagues developed multiple machine learning prediction models for Bethesda category III thyroid nodules, which are clinically challenging to differentiate (14). This study enrolled 2,500 patients and compared 10 different algorithms, with logistic regression achieving an AUC of 0.823 on the validation set (14). XGBoost models have demonstrated significant success in thyroid cancer prediction (15, 16). Li and colleagues integrated clinical, ultrasound, and thyroid function indices, with the XGBoost model achieving an AUC of 0.928 and accuracy of 0.851 (15). Ozturk and colleagues achieved 95.3% accuracy and 98.2% AUC using XGBoost (16).

Deep learning has also demonstrated important applications in thyroid imaging analysis (17). Zhang and colleagues proposed a deep learning model based on dynamic ultrasound video that achieved AUC values of 0.947 and 0.923 in the training and validation cohorts, respectively (17). Furthermore, multiple studies have explored the impact of different feature selection methods on model performance (18). Thompson and colleagues further elucidated this issue in interpretable ensemble methods (18). Compared with single feature selection algorithms, the intersection of multiple feature selection methods can more effectively screen out truly important features, reduce false discovery rate, and enhance model reproducibility across different datasets (18). This methodological advance is critical for establishing robust and reproducible prediction models (18, 19). Kim and colleagues further validated the effectiveness of this strategy using ultrasound-guided nomograms (19).

### Summary and advantages of the present study

The present study addresses the clinical need for improved prediction of distant metastasis in PTC and the limitations of existing research by establishing a comprehensive prediction model integrating clinical features, laboratory indices, and ultrasound report characteristics. Compared with existing studies, the present research possesses the following innovations and advantages.

First, the study employed a multilevel data processing and feature selection approach to ensure model robustness and credibility. Specifically, this study applied natural language processing techniques to automatically extract key features from ultrasound reports (such as nodule echogenicity, boundary clarity, and blood flow signals), substantially reducing human bias and improving data processing efficiency compared with manual coding methods—a development of particular importance for large-scale clinical applications. Second, the study utilized the intersection of three feature selection methods (LASSO, RFE, and ReliefF) rather than relying on a single algorithm, further enhancing the credibility of feature screening. Pu and colleagues demonstrated that selecting 10 key features from 615 total features, the LightGBM model achieved an AUC of 0.996, confirming the effectiveness of multi-algorithm fusion (2).

Third, the study systematically compared the performance of multiple algorithms and integrated interpretability analysis, making model decisions more transparent. The present research compared the performance of nine mainstream machine learning algorithms (logistic regression, naïve Bayes, decision tree, gradient boosting, random forest, multilayer perceptron, XGBoost, LightGBM, and K-nearest neighbors) and systematically evaluated the applicability of each algorithm in PTC metastasis prediction, providing empirical support for algorithm selection. Most importantly, the study integrated SHAP (SHapley Additive exPlanations) interpretability analysis, providing not only prediction results but also revealing the model’s decision logic, enabling clinicians to understand the contribution of each feature to metastasis risk. Redlich and colleagues confirmed the effectiveness of the XGBoost + SHAP approach, with this study deepening its application (20).

Fourth, the study comprehensively integrated multidimensional data and developed prediction tools suitable for clinical practice. The study comprehensively incorporated key immunological indices such as thyroid-stimulating hormone (TSH), thyroglobulin antibody (TgAb), and thyroid peroxidase antibody (TPOAb), rather than relying solely on clinical or imaging features. This multidimensional data fusion more comprehensively reflects the biological characteristics of PTC. Johnson and colleagues emphasized the importance of multidimensional data in their integration study of circulating tumor cells (21). Finally, the study designed and developed a user-friendly prediction website and software, enabling the model to be readily employed by clinicians and promoting the translation of AI research from laboratory to clinical practice.

## Research methods

### Medical record collection

#### Inclusion criteria

Patients enrolled in the study were required to meet all of the following criteria:

Pathological confirmation: Pathological histological or cytological confirmation of papillary thyroid carcinoma (PTC).

Treatment timeframe: Diagnosed and underwent initial treatment (including total thyroidectomy or near-total thyroidectomy) between 2015 and 2023.

Age requirement: Adult patients aged ≥18 years.

Data completeness: Complete clinical, pathological, laboratory examination, and follow-up data.

Follow-up requirement: At least 6 months or longer of follow-up records after initial treatment documenting metastasis occurrence.

#### Exclusion criteria

Patients in the following situations were excluded:

Pathology-related: Non-PTC thyroid malignancies (such as anaplastic carcinoma, medullary carcinoma, thyroid lymphoma, etc.) or benign nodules.

Missing clinical information: Absence of key clinical features (such as TNM stage information or initial diagnostic ultrasound report); absence of critical laboratory indices (such as TSH, TgAb, TPOAb, etc.).

Follow-up related: Loss to follow-up or insufficient follow-up duration of less than 6 months; patients in whom distant metastasis status could not be determined.

Other factors: Pregnant or nursing patients; patients with history of other malignancies; patients with severe organ dysfunction affecting treatment.

#### Data collection scope

Complete medical records were collected for patients meeting inclusion/exclusion criteria, including:

Demographic data: Age, sex, body mass index (BMI).

Clinicopathological features: TNM stage, tumor size, multifocality, thyroid capsule invasion, extrathyroidal extension, neural invasion, pathological type, and degree of malignancy.

Imaging reports: Ultrasound reports including nodule size, number of nodules, nodule status (cystic-solid composition, etc.), echo characteristics (isoechoic, hypoechoic, etc.), boundary clarity, calcification, and blood flow signal intensity.

Laboratory investigations:

Thyroid function indices: Thyroid-stimulating hormone (TSH), free thyroxine (FT4).

Tumor markers: Serum thyroglobulin (Tg), thyroglobulin antibody (TgAb).

Immunological indices: Thyroid peroxidase antibody (TPOAb), neutrophil-to-lymphocyte ratio (NLR), platelet-to-lymphocyte ratio (PLR), etc.

Follow-up data:

Distant metastasis occurrence: Specific sites and timing of bone, lung, brain, and other visceral metastases.

Time to metastasis: Time interval from initial treatment to diagnosis of distant metastasis.

Follow-up outcomes: Patient status at last follow-up (no distant metastasis/metastasis occurred) and follow-up duration.

### Database establishment and feature extraction

A PostgreSQL database was established for structured data management. Patient tables, examination result tables, and follow-up tables were designed to ensure data integrity and traceability. Automated scripts extracted numerical features, including age and various laboratory indices. Natural language processing technology was utilized to extract key features from non-structured ultrasound reports.

### Feature selection

A three-level progressive feature selection strategy was employed. The first step separately applied three feature selection methods: LASSO regression (Least Absolute Shrinkage and Selection Operator) for automatic selection of important features through L1 regularization, Recursive Feature Elimination (RFE) for iterative removal of the least important features, and ReliefF algorithm for feature weight evaluation based on instance distance. The second step extracted the intersection of the top 15 features from the three methods, ensuring that selected features demonstrated high importance across multiple algorithms. The third step extracted the intersection of important features as model input.

### Model development and class imbalance handling

In the development cohort, we randomly split the data into training and internal test sets using a 70:30 ratio with a fixed random seed (random_state = 42) to ensure reproducibility. Because of the substantial imbalance between metastatic and non-metastatic patients, Synthetic Minority Over-sampling Technique (SMOTE) was applied only to the training set to balance the class distribution before model training.Then nine widely used machine learning algorithms were used to construct prediction models: LogisticRegression (max_iter = 5000, random_state = 42), GaussianNB(), DecisionTreeClassifier (random_state = 42), GradientBoostingClassifier (random_state = 42), RandomForestClassifier (random_state = 42, n_jobs = −1), MLPClassifier (random_state = 42, max_iter = 5000), XGBClassifier (random_state = 42, n_jobs = −1), LGBMClassifier (random_state = 42, n_jobs = −1), and KNeighborsClassifier(). Model performance was evaluated using AUC, accuracy, sensitivity, specificity, and F1-score. The optimal model was selected based on comprehensive multi-metric evaluation. Hyperparameter tuning was performed on the optimal model.

### SHAP interpretability analysis

After model comparison, the LightGBM (LGBM) classifier, which achieved the highest AUC among all candidates, was selected as the optimal model for further interpretation. We then applied the SHAP (SHapley Additive exPlanations) TreeExplainer to the final LGBM model to quantify feature contributions at both the global and individual levels. The mean absolute SHAP value (Mean |SHAP|) and mean SHAP value (Mean SHAP) for each feature were calculated to reflect directional influence on model prediction. Feature importance ranking and local explanation plots were generated to enhance model credibility and clinical application value.

### External validation and Django-based prediction website and software development

Medical records from 421 papillary thyroid carcinoma patients, including both metastatic(82) and non−metastatic cases(329), were collected from Jimo District People’s Hospital, a secondary care hospital with a different catchment area than the development center, to serve as an independent external validation cohort. Patient selection followed the same inclusion and exclusion criteria as in the development cohort, and key baseline characteristics (age, sex, TNM stage, tumor size, and major laboratory indices such as TSH, TgAb, and TPOAb).

Based on the optimal model and its SHAP-based interpretation results, we implemented an interactive, web-based prototype for PTC distant metastasis prediction using the Django framework as a proof-of-concept clinical decision support tool.

## Results

### Demographic characteristics

**Figure 1:**
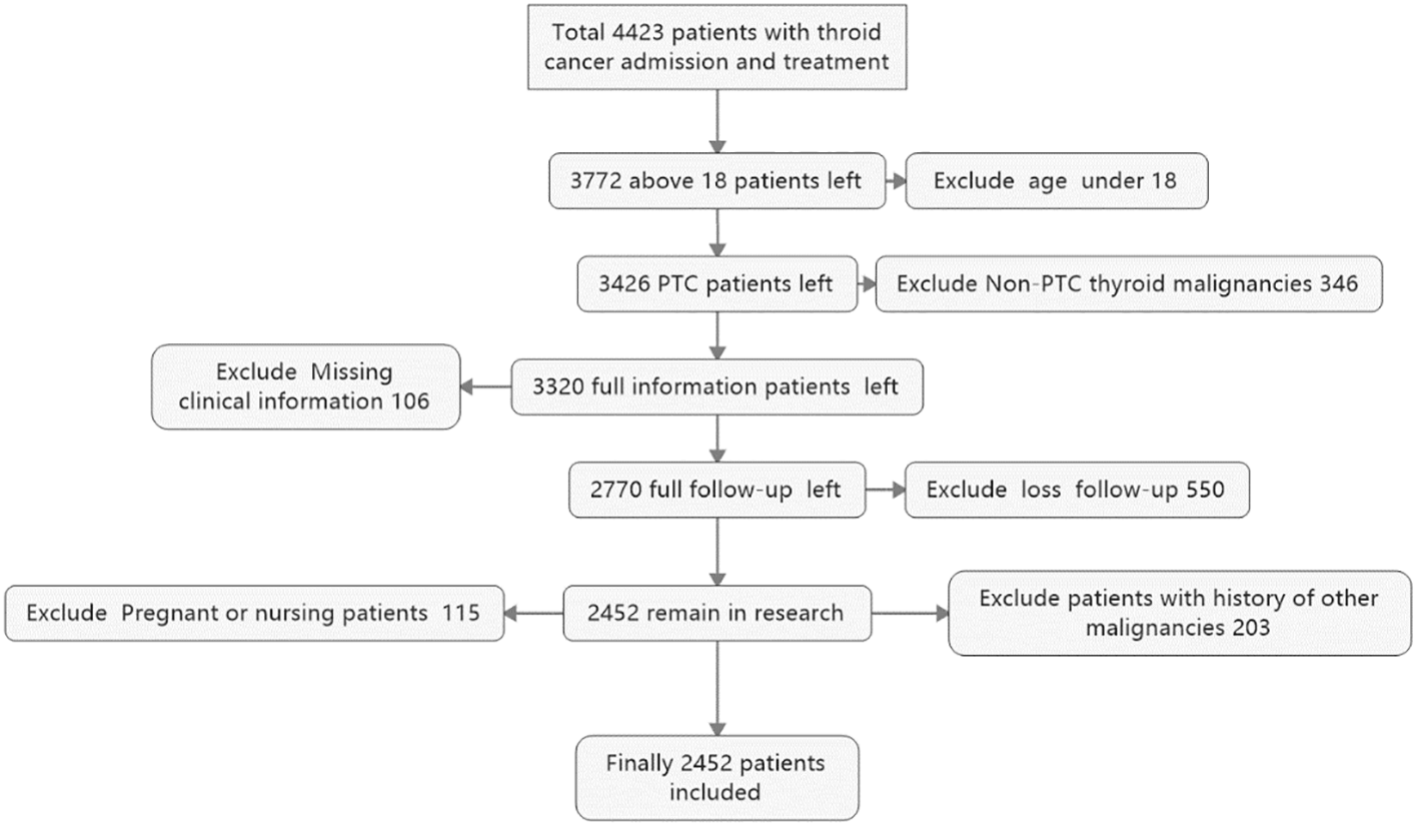
Flowchart of patient inclusion and exclusion for the development and external validation cohorts. The diagram summarizes the inclusion and exclusion of patients for the development cohort (Qilu Hospital) and the external validation cohort (Jimo District People’s Hospital).

A total of 2,452 thyroid cancer patients were enrolled in the study as the effective sample, selection procedure is in **Figure 1**. Patients were stratified into two groups based on metastasis status: non-metastasis group (n = 1,996, 81.4%) and metastasis group (n = 456, 18.6%), statistical results is in **Table 1**. Normality testing (Shapiro-Wilk test) and homogeneity of variance testing (Levene test) were employed to determine the appropriate statistical comparison method for continuous variables. Continuous variables demonstrating normal distribution and homogeneous variance were analyzed using independent samples t test; non-normally distributed variables were analyzed using Mann-Whitney U test. Categorical variables were analyzed using chi-square test (expected frequency ≥5) or Fisher exact test (expected frequency <5), with statistical significance set at α = 0.05. Results demonstrated that among continuous variables, tumor size (metastasis group vs. non-metastasis group: 3.912 ± 1.187 cm vs. 2.405 ± 0.913 cm; t = −15.623; *p* < 0.001) and age (metastasis group vs. non-metastasis group: 53.124 ± 11.356 years vs. 45.789 ± 10.521 years; t = −8.975; *p* < 0.001) showed highly significant differences between groups. Among categorical variables, T stage (χ² = 58.231; *p* < 0.001), N stage (χ² = 92.456; *p* < 0.001), multifocality (χ² = 36.789; *p* < 0.001), extrathyroidal invasion (χ² = 49.123; *p* < 0.001), calcification (χ² = 32.567; *p* < 0.001), degree of malignancy (χ² = 55.890; *p* < 0.001), positive molecular detection (OR = 4.872; *p* < 0.001), and thyroid dysfunction (χ² = 22.345; *p* < 0.001) were all significantly associated with thyroid cancer metastasis. In contrast, body weight, BMI, sex, and smoking history showed no significant between-group differences (*p* > 0.05), suggesting that the aforementioned significant factors may be associated with metastasis risk in thyroid cancer and could serve as candidate variables for subsequent multivariate regression analysis (22).

**Table 1:** Comparison of baseline clinical and imaging characteristics between papillary thyroid cancer patients with and without metastasis.

Variable_Name	Variable_Type	No_Metastasis (Mean ± SD)	Metastasis (Mean ± SD)	Test_Method	Test_Statistic	P_Value	Significance	No_Metastasis (Count%)	Metastasis (Count%)
border_sharpness	Continuous	1.549 ± 0.550	1.326 ± 0.496	Mann-Whitney U test	551126	0	***		
Anti-ThyroidAntibody	Continuous	9.468 ± 53.712	7.021 ± 40.398	Mann-Whitney U test	599906	0	***		
TgAb	Continuous	175.439 ± 45.259	193.058 ± 47.982	Mann-Whitney U test	289884	0	***		
Gender	Categorical			Chi-square test	132.728 (df=1)	0	***	0: 1250 (62.6%) 1: 746 (37.4%)	0: 150 (32.9%) 1: 306 (67.1%)
NoduleStatus	Categorical			Chi-square test	25.045 (df=2)	0	***	0: 633 (31.7%) 1: 1169 (58.6%) -1: 194 (9.7%)	0: 95 (20.8%) 1: 295 (64.7%) -1: 66 (14.5%)
ExtrathyroidalInvasion	Categorical			Chi-square test	136.303 (df=1)	0	***	0: 1171 (58.7%) 1: 825 (41.3%)	0: 129 (28.3%) 1: 327 (71.7%)
Recurrence	Categorical			Chi-square test	157.613 (df=1)	0	***	0: 1951 (97.7%) 1: 45 (2.3%)	0: 381 (83.6%) 1: 75 (16.4%)
subtype	Categorical			Fisher's exact test	1.22237E-39	0.0001	***	TallCellVariant: 21 (1.1%) FollicularVariant: 11 (0.6%) DiffuseSclerosisVariant: 11 (0.6%) ClassicalVariant: 1953 (97.8%)	TallCellVariant: 75 (16.4%) FollicularVariant: 1 (0.2%) DiffuseSclerosisVariant: 1 (0.2%) ClassicalVariant: 379 (83.1%)
density	Categorical			Fisher's exact test	1.17213E-12	0.0001	***	1.2: 1 (0.1%) 1.0: 1194 (59.8%) 2.0: 761 (38.1%) 3.0: 22 (1.1%) 1.1: 17 (0.9%) 1.3: 1 (0.1%)	1.2: 3 (0.7%) 1.0: 218 (47.8%) 2.0: 219 (48.0%) 3.0: 2 (0.4%) 1.1: 11 (2.4%) 1.3: 3 (0.7%)
ThyroidFunction	Categorical			Chi-square test	14.663 (df=1)	0.0001	***	0: 296 (14.8%) 1: 1700 (85.2%)	0: 36 (7.9%) 1: 420 (92.1%)
TPOAb	Continuous	786.576 ± 204.796	780.767 ± 184.970	Mann-Whitney U test	505268	0.0002	***		
Age	Continuous	39.517 ± 11.814	41.561 ± 11.842	Mann-Whitney U test	409258	0.0008	***		
Calcification	Categorical			Fisher's exact test	6.75718E-09	0.0017	**	0: 178 (8.9%) 1: 604 (30.3%) 2: 899 (45.0%) 3: 299 (15.0%) 4: 16 (0.8%)	0: 26 (5.7%) 1: 132 (28.9%) 2: 193 (42.3%) 3: 97 (21.3%) 4: 8 (1.8%)
enhancementDegree	Continuous	3.196 ± 0.904	3.316 ± 0.921	Mann-Whitney U test	415771.5	0.0019	**		
BloodFlowSignal	Categorical			Fisher's exact test	5.50726E-07	0.0067	**	0: 1324 (66.3%) 1: 31 (1.6%) 2: 569 (28.5%) 3: 68 (3.4%) 4: 4 (0.2%)	0: 280 (61.4%) 1: 1 (0.2%) 2: 151 (33.1%) 3: 24 (5.3%) 4: 0 (0.0%)
SerumMarkers	Categorical			Chi-square test	5.405 (df=1)	0.0201	*	0: 607 (30.4%) 1: 1389 (69.6%)	0: 113 (24.8%) 1: 343 (75.2%)
TStage	Categorical			Chi-square test	8.117 (df=3)	0.0436	*	1: 1871 (93.7%) 2: 64 (3.2%) 3: 34 (1.7%) 4: 27 (1.4%)	1: 437 (95.8%) 2: 4 (0.9%) 3: 10 (2.2%) 4: 5 (1.1%)
Multifocality	Categorical			Chi-square test	2.826 (df=1)	0.0927	ns	0: 1451 (72.7%) 1: 545 (27.3%)	0: 313 (68.6%) 1: 143 (31.4%)
AlcoholConsumptionHistory	Categorical			Chi-square test	2.516 (df=1)	0.1127	ns	0: 1918 (96.1%) 1: 78 (3.9%)	0: 430 (94.3%) 1: 26 (5.7%)
Long-termMedicationHistory	Categorical			Chi-square test	2.468 (df=1)	0.1162	ns	0: 1925 (96.4%) 1: 71 (3.6%)	0: 447 (98.0%) 1: 9 (2.0%)
position	Categorical			Fisher's exact test	1.14361E-08	0.1572	ns	RightLobeAndIsthmus: 20 (1.0%) LeftLobe: 703 (35.2%) IsthmusNearLeftLobe: 4 (0.2%) Multifocality: 417 (20.9%) BothLobes: 19 (1.0%) RightLobe: 812 (40.7%) Isthmus: 10 (0.5%) RightSideLobe: 4 (0.2%) BilateralLobes: 7 (0.4%)	RightLobeAndIsthmus: 4 (0.9%) LeftLobe: 169 (37.1%) IsthmusNearLeftLobe: 0 (0.0%) Multifocality: 111 (24.3%) BothLobes: 1 (0.2%) RightLobe: 164 (36.0%) Isthmus: 6 (1.3%) RightSideLobe: 0 (0.0%) BilateralLobes: 1 (0.2%)
Echogenicity	Categorical			Fisher's exact test	2.18855E-05	0.1915	ns	0: 1946 (97.5%) 1: 4 (0.2%) 2: 7 (0.4%) 3: 4 (0.2%) 4: 4 (0.2%) 5: 19 (1.0%) 6: 4 (0.2%) 7: 8 (0.4%)	0: 450 (98.7%) 1: 0 (0.0%) 2: 5 (1.1%) 3: 0 (0.0%) 4: 0 (0.0%) 5: 1 (0.2%) 6: 0 (0.0%) 7: 0 (0.0%)
Calcification_1	Categorical			Chi-square test	2.873 (df=2)	0.2378	ns	0: 1671 (83.7%) 1: 99 (5.0%) 2: 226 (11.3%)	0: 377 (82.7%) 1: 17 (3.7%) 2: 62 (13.6%)
AssociatedSymptoms	Categorical			Chi-square test	1.359 (df=1)	0.2437	ns	0: 1973 (98.8%) 1: 23 (1.2%)	0: 447 (98.0%) 1: 9 (2.0%)
Comorbidities	Categorical			Chi-square test	1.345 (df=1)	0.2462	ns	0: 1903 (95.3%) 1: 93 (4.7%)	0: 441 (96.7%) 1: 15 (3.3%)
NStage	Categorical			Chi-square test	1.260 (df=1)	0.2616	ns	0: 45 (2.3%) 1: 1951 (97.7%)	0: 15 (3.3%) 1: 441 (96.7%)
TumorSize	Continuous	4.902 ± 6.904	4.753 ± 6.677	Mann-Whitney U test	444780	0.4494	ns		
MalignancyGrade	Categorical			Chi-square test	2.394 (df=3)	0.4947	ns	0: 39 (2.0%) 4: 1601 (80.2%) 5: 286 (14.3%) 6: 70 (3.5%)	0: 9 (2.0%) 4: 355 (77.9%) 5: 78 (17.1%) 6: 14 (3.1%)
HistoryofOtherMalignantTumors	Categorical			Fisher's exact test	0.396603397	0.7077	ns	0: 1985 (99.4%) 1: 11 (0.6%)	0: 455 (99.8%) 1: 1 (0.2%)
PreviousThyroidDiseaseHistory	Categorical			Chi-square test	0.119 (df=1)	0.7297	ns	0: 1972 (98.8%) 1: 24 (1.2%)	0: 452 (99.1%) 1: 4 (0.9%)
Morphology	Categorical			Chi-square test	0.103 (df=1)	0.7485	ns	0: 472 (23.6%) 1: 1524 (76.4%)	0: 104 (22.8%) 1: 352 (77.2%)
TumorDetectionMethod	Categorical			Chi-square test	0.063 (df=1)	0.8018	ns	0: 1971 (98.7%) 1: 25 (1.3%)	0: 449 (98.5%) 1: 7 (1.5%)
SmokingHistory	Categorical			Chi-square test	0.011 (df=1)	0.9164	ns	0: 1909 (95.6%) 1: 87 (4.4%)	0: 435 (95.4%) 1: 21 (4.6%)
BMI	Continuous	24.913 ± 4.364	24.970 ± 3.805	Mann-Whitney U test	456400	0.9232	ns		
Weight	Continuous	65.046 ± 20.840	64.838 ± 20.618	Mann-Whitney U test	455138	0.9971	ns		
FamilyGeneticHistory	Categorical			Fisher's exact test	0	1	ns	0: 1992 (99.8%) 1: 4 (0.2%)	0: 456 (100.0%) 1: 0 (0.0%)
MolecularTestingResult	Categorical			Fisher's exact test	0	1	ns	0: 1992 (99.8%) 1: 4 (0.2%)	0: 456 (100.0%) 1: 0 (0.0%)
PreviousHeadRadiationTherapyHistory	Categorical			Chi-square test	0.000 (df=0)	1	ns	0: 1996 (100.0%)	0: 456 (100.0%)
MStage	Categorical			Fisher's exact test	0	1	ns	0: 1992 (99.8%) 1: 4 (0.2%)	0: 456 (100.0%) 1: 0 (0.0%)

This table compares clinical data, laboratory markers, and ultrasound imaging features between the non-metastasis group and metastasis group. Continuous variables were analyzed using the Mann-Whitney U test, while categorical variables were compared using the chi-square test or Fisher’s exact test. The table presents the corresponding test statistics, P-values, and significance indicators (***p<0.001, **p<0.01, *p<0.05, ns=not significant).

### Feature selection

**Figure 2:**
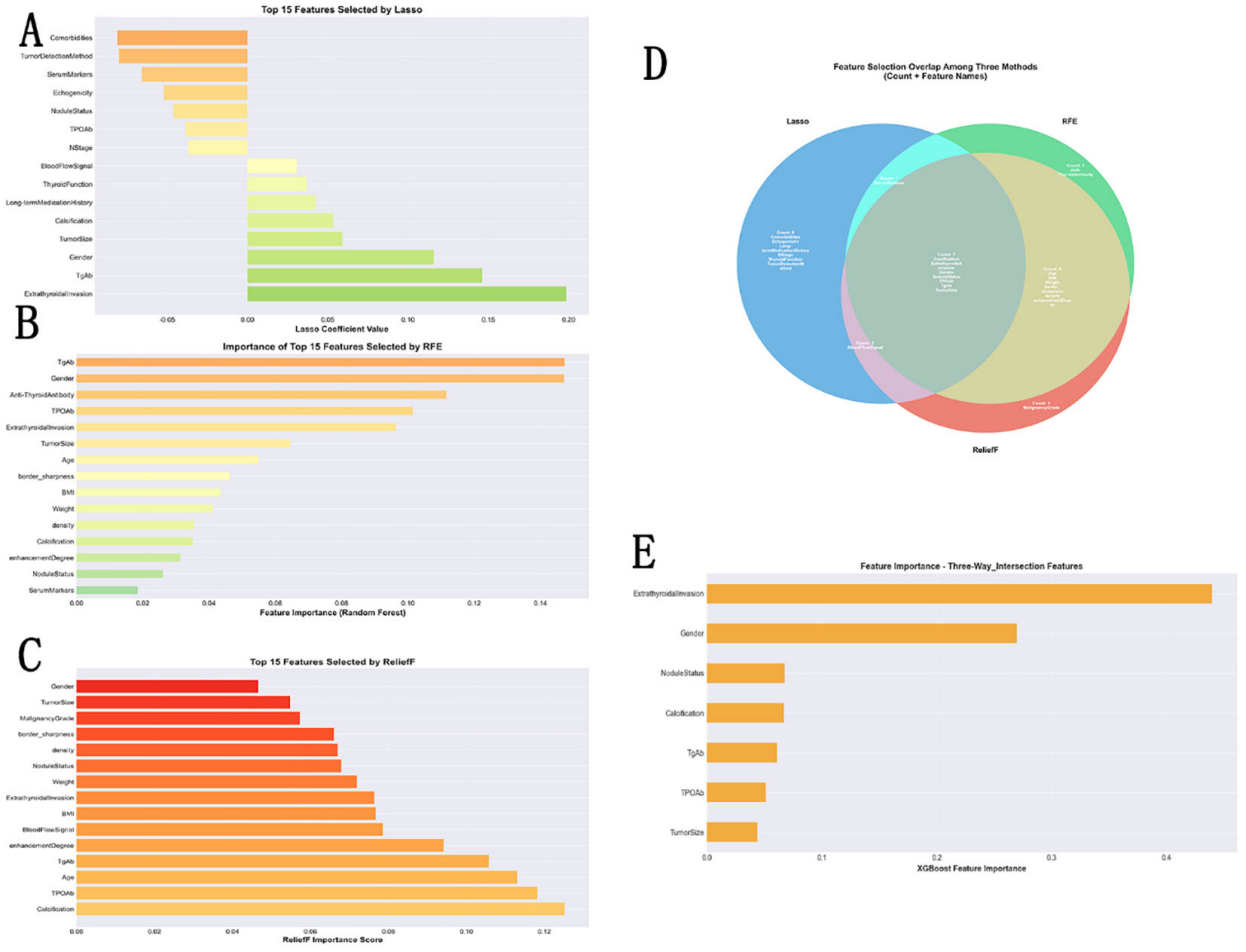
Feature selection for PTC distant metastasis prediction: LASSO, RFE, and ReliefF​. **(A–C)** Feature importance ranking obtained with three complementary methods: LASSO, recursive feature elimination (RFE), and ReliefF. **(D)** Venn diagram showing the overlap of the top 15 features from each method; seven features are consistently selected by all three algorithms. **(E)** Final ranking of these seven core features, which are subsequently used for model development to reduce redundancy, limit overfitting, and enhance clinical interpretability.

Feature selection utilized “Metastasis” as the target variable, based on the original dataset with dimensions of (2452, 40) containing 36 features. Data preprocessing was first completed, with 456 positive samples and 1,996 negative samples (positive sample proportion: 18.6%); the resampled training set contained 2,514 samples and the test set contained 736 samples. Three feature selection methods—LASSO, RFE (fixed retention of top 15 features), and ReliefF—each selected 15 features. Intersection analysis revealed seven features commonly selected by all three methods: [Calcification, ExtrathyroidalInvasion, Gender, NoduleStatus, TPOAb, TgAb, TumorSize]. Additionally, fifteen features were selected by at least two methods (loose intersection), encompassing key indices such as age, BMI, and blood flow signal. Furthermore, LASSO uniquely selected 6 features, while RFE and ReliefF each uniquely selected 1 feature; the intersection of RFE and ReliefF yielded 6 features. The three methods combined identified 23 unique features, providing the core feature basis for subsequent development of metastasis prediction models,. Feature Selection analysis and results is in **Figure 2**.

### Model performance comparison

**Figure 3:**
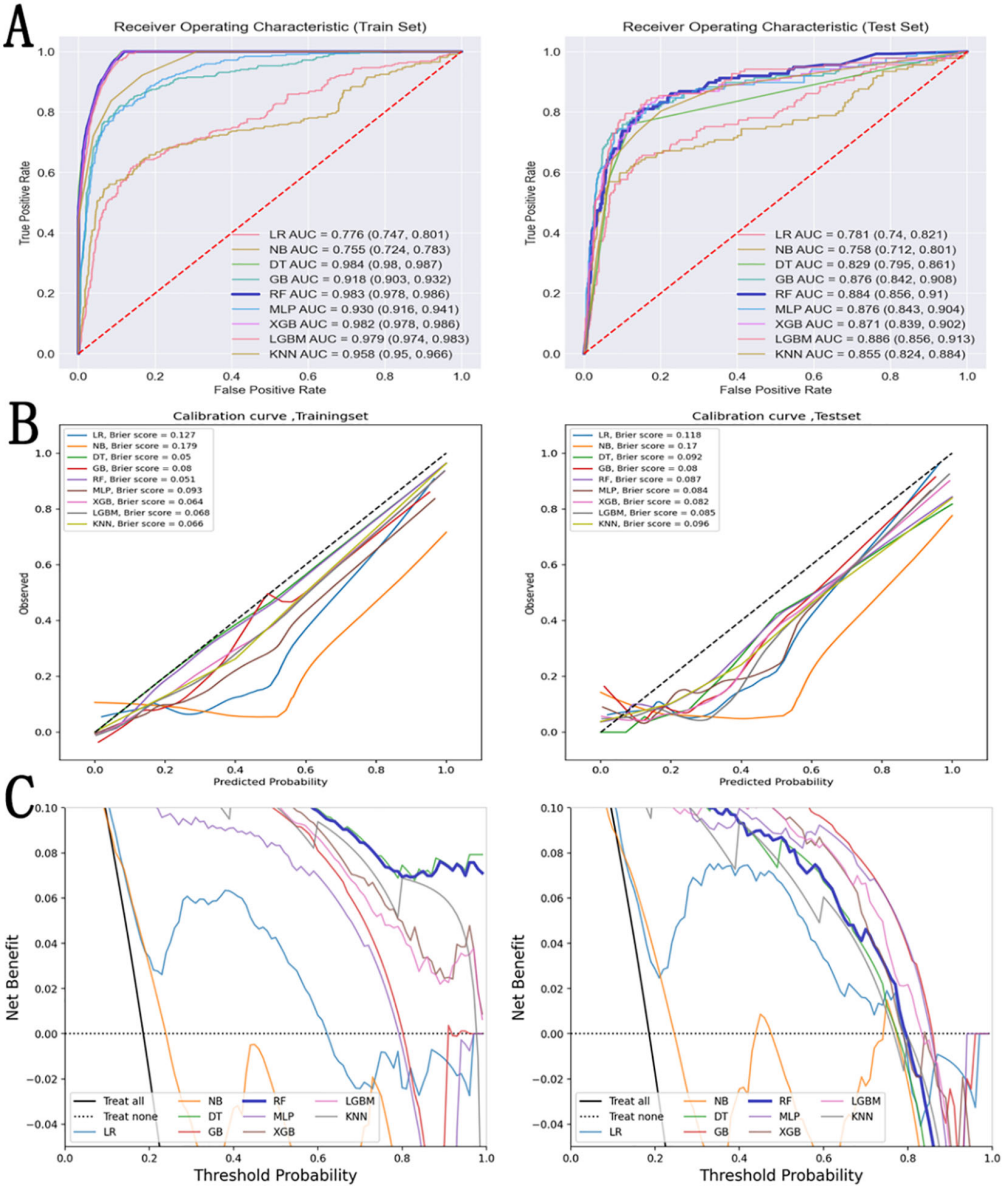
Performance comparison of 9 machine learning models for PTC distant metastasis prediction​. **(A)** ROC curves and AUC values for nine models (LR, GNB, DT, RF, GB, MLP, KNN, XGB, LGBM) in the training and internal test sets, showing superior discrimination of the LightGBM model. **(B)** Calibration curves and Brier scores in the internal test set, indicating that LightGBM provides the best agreement between predicted and observed metastasis risk. **(C)** Decision curve analysis comparing net clinical benefit of the different models across a range of threshold probabilities, with LightGBM yielding the highest net benefit relative to “treat-all” and “treat-none” strategies.

Nine machine learning models were constructed based on the selected features: logistic regression (LR), naïve Bayes (NB), decision tree (DT), gradient boosting (GB), random forest (RF), multilayer perceptron (MLP), XGBoost (XGB), LightGBM (LGBM), and K-nearest neighbors (KNN). Model performance was evaluated primarily using test set metrics (metric values formatted as “training set | test set”), with comprehensive assessment using AUC, accuracy, recall rate (sensitivity), specificity, and F1-score and all the results are in (**Figure 3**, **Table 2**). Overall, ensemble learning and tree-based models demonstrated substantially superior test set performance compared with traditional linear models. LightGBM demonstrated optimal test set performance with AUC of 0.886 and accuracy of 0.887, recall rate of 0.723, and F1-score of 0.705, with balanced metrics and no significant weaknesses. Random forest and XGBoost followed closely with test set AUC values of 0.884 and 0.871, respectively, and accuracy around 0.87, with recall rates between 0.66–0.68, and specificity maintained at 0.91–0.92, demonstrating stable performance in both identifying metastatic cases and excluding non-metastatic cases. Gradient boosting, multilayer perceptron, and K-nearest neighbors demonstrated intermediate performance, with test set AUC concentrated at 0.85–0.88 and accuracy of 0.84–0.88; notably, KNN achieved a test set recall rate of 0.730, although precision was relatively low (0.552). Among traditional models, LR demonstrated test set AUC of 0.781 and accuracy of 0.799 with recall rate of 0.657, showing moderate performance. NB demonstrated the highest test set specificity (0.883) but the lowest recall rate (0.606), indicating weaker ability in identifying positive samples. Decision tree exhibited marked overfitting, with training set AUC of 0.984 and accuracy of 0.935, while test set AUC dropped sharply to 0.829 with accuracy of 0.868 and recall rate of only 0.584, demonstrating insufficient generalization ability. In summary, LGBM, RF, and XGB models demonstrated optimal comprehensive test set performance with superior generalization ability and were therefore selected as the final prediction models.

**Table 2:** Classification performance of multiple machine learning models.

Model	AUC	Accuracy	Sensitivity/Recall	Specificity	F1-score	PPV/precision	NPV
LR	0.776 | 0.781	0.797 | 0.799	0.633 | 0.657	0.834 | 0.831	0.537 | 0.549	0.465 | 0.471	0.909 | 0.914
NB	0.755 | 0.758	0.821 | 0.832	0.577 | 0.606	0.877 | 0.883	0.545 | 0.572	0.517 | 0.542	0.901 | 0.907
DT	0.984 | 0.829	0.935 | 0.868	0.755 | 0.584	0.976 | 0.933	0.813 | 0.623	0.880 | 0.667	0.946 | 0.907
GB	0.918 | 0.876	0.902 | 0.882	0.768 | 0.730	0.932 | 0.917	0.744 | 0.697	0.721 | 0.667	0.946 | 0.937
RF	0.983 | 0.884	0.935 | 0.871	0.828 | 0.679	0.960 | 0.915	0.826 | 0.662	0.825 | 0.646	0.961 | 0.926
MLP	0.930 | 0.876	0.890 | 0.872	0.768 | 0.745	0.918 | 0.902	0.723 | 0.685	0.682 | 0.634	0.945 | 0.939
XGB	0.982 | 0.871	0.935 | 0.870	0.831 | 0.664	0.958 | 0.917	0.826 | 0.655	0.820 | 0.645	0.961 | 0.923
LGBM	0.979 | 0.886	0.931 | 0.887	0.818 | 0.723	0.957 | 0.925	0.816 | 0.705	0.813 | 0.688	0.958 | 0.936
KNN	0.958 | 0.855	0.900 | 0.840	0.837 | 0.730	0.915 | 0.865	0.806 | 0.615	0.692 | 0.552	0.961 | 0.933

This table demonstrates the predictive performance of various machine learning models (Logistic Regression, Naive Bayes, Decision Tree, Gradient Boosting, Random Forest, Multi-Layer Perceptron, XGBoost, LightGBM, and K-Nearest Neighbors) on both training and validation/test sets. Performance metrics include Area Under the Receiver Operating Characteristic Curve (AUC), accuracy, sensitivity/recall, specificity, F1-score, positive predictive value (PPV/precision), negative predictive value (NPV), and optimal classification cutoff values, enabling comprehensive comparison of model discrimination ability.

### SHAP interpretability analysis

**Figure 4:**
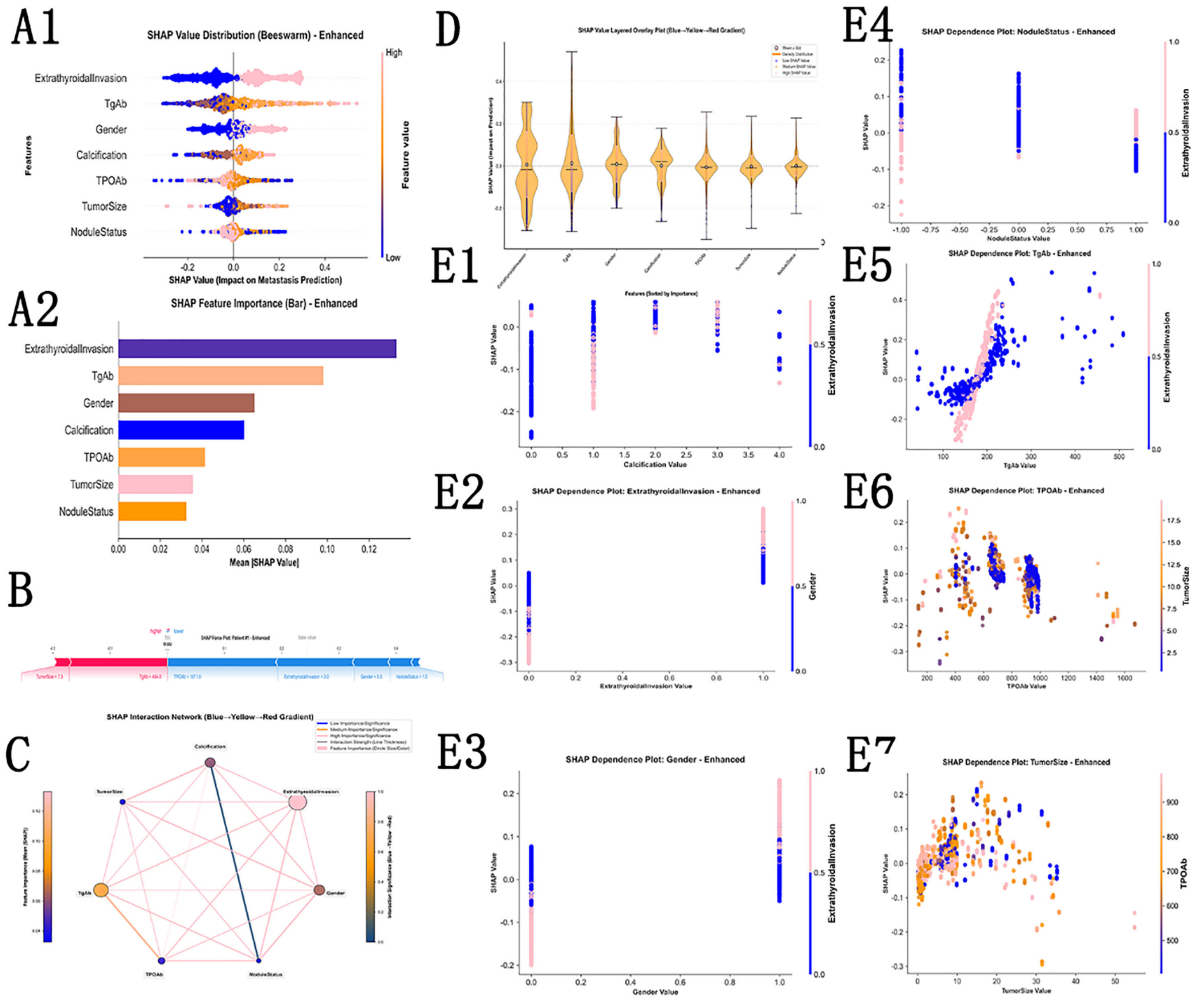
SHAP-based interpretability of the LightGBM model for PTC distant metastasis prediction​. **(A)** Global SHAP summary plots (beeswarm and bar plot) showing the relative importance and directional effects of the seven core features (Calcification, ExtrathyroidalInvasion, Gender, NoduleStatus, TPOAb, TgAb, TumorSize) on metastasis prediction. **(B)** Representative local explanation (force plot) illustrating how individual feature values increase or decrease the predicted metastasis risk for a single patient. **(C)** SHAP dependence plots demonstrating nonlinear and interaction effects (e.g., for tumor size and antibody indices) on model output, providing insight into complex risk patterns beyond simple linear associations. **(D)** SHAP value layered density (violin) plot displaying the distribution, density, and spread of SHAP values for each of the seven core features across all patients, with a blue–yellow–red gradient indicating low-to-high feature values, illustrating both the magnitude and direction of each feature's contribution to distant metastasis prediction. **(E1)** SHAP dependence plot for Calcification, showing the relationship between calcification category values (0–4) and their corresponding SHAP values, with color coding indicating the interacting feature ExtrathyroidalInvasion; higher calcification grades are associated with increased positive SHAP contributions to metastasis risk. **(E2)** SHAP dependence plot for ExtrathyroidalInvasion, demonstrating a sharp positive shift in SHAP values when extrathyroidal invasion is present (value = 1), with Gender as the interacting feature, confirming extrathyroidal invasion as the strongest individual predictor of distant metastasis. **(E3)** SHAP dependence plot for Gender, illustrating the differential SHAP contribution between female (0) and male (1) patients, with ExtrathyroidalInvasion as the color-coded interacting feature, indicating that male sex is associated with higher metastasis risk contribution. **(E4)** SHAP dependence plot for NoduleStatus, showing how single, multifocal, and bilateral nodule configurations (–1, 0, 1) relate to SHAP values, with ExtrathyroidalInvasion as the interacting feature, revealing that multifocal nodule status confers a modestly elevated metastasis risk. **(E5)** SHAP dependence plot for TgAb, demonstrating a nonlinear positive association between serum thyroglobulin antibody levels and SHAP values across the observed range, with ExtrathyroidalInvasion as the interacting feature; higher TgAb values correspond to progressively greater positive contributions to predicted metastasis risk. **(E6)** SHAP dependence plot for TPOAb, illustrating the complex, nonlinear relationship between thyroid peroxidase antibody levels and their SHAP contributions, color-coded by TumorSize as the interacting feature, indicating that the direction and magnitude of TPOAb’s effect on metastasis risk varies across its value range. **(E7)** SHAP dependence plot for TumorSize, showing the nonlinear association between primary tumor diameter (cm) and SHAP values, with TPOAb as the color-coded interacting feature; the pattern suggests that the relationship between tumor size and metastasis risk is not strictly monotonic, supporting a complex biological interaction rather than a simple linear effect.

SHAP interpretability analysis was conducted based on the seven core metastasis prediction features identified previously ([Calcification, ExtrathyroidalInvasion, Gender, NoduleStatus, TPOAb, TgAb, TumorSize]), using a standardized dataset with dimensions of (2452, 7) and showed in **Figure 4**. During the analysis, SHAP values matching the sample size and number of features were successfully calculated (dimensions: (2452, 7)), with numerical range from −0.3344 to −0.5424 and mean absolute SHAP value of 0.0665, reflecting moderate overall impact of features on model prediction. To comprehensively interpret feature contributions, multiple key visualization plots were generated: SHAP beeswarm plot (01_shap_beeswarm.png) and violin plot (02_shap_violin.png) directly present the distribution characteristics of SHAP values for each feature and their associations with prediction results, clearly demonstrating the positive and negative directional impacts of different features on metastasis risk. The feature importance bar plot (03_shap_bar.png) quantifies the contribution weight of the seven core features, providing basis for screening more critical prediction factors. For the first sample (predicted metastasis probability: 0.0005, classified as non-metastatic, with feature values including calcification = 1.0, no extrathyroidal invasion, female sex, nodule status = 1.0, etc.), a force plot (04_shap_force_sample_1.png) was generated to visualize the driving or inhibitory effects of each feature on the prediction result for this sample. Waterfall plots for the top five samples further decomposed the cumulative contribution of features in individual predictions. Overall, SHAP analysis not only clarified the magnitude and direction of impact of core features on thyroid cancer metastasis prediction, but also achieved interpretability coverage from the overall model to individual samples, substantially enhancing the credibility and clinical application value of the prediction model.

### Django prediction website development

**Figure 5:**
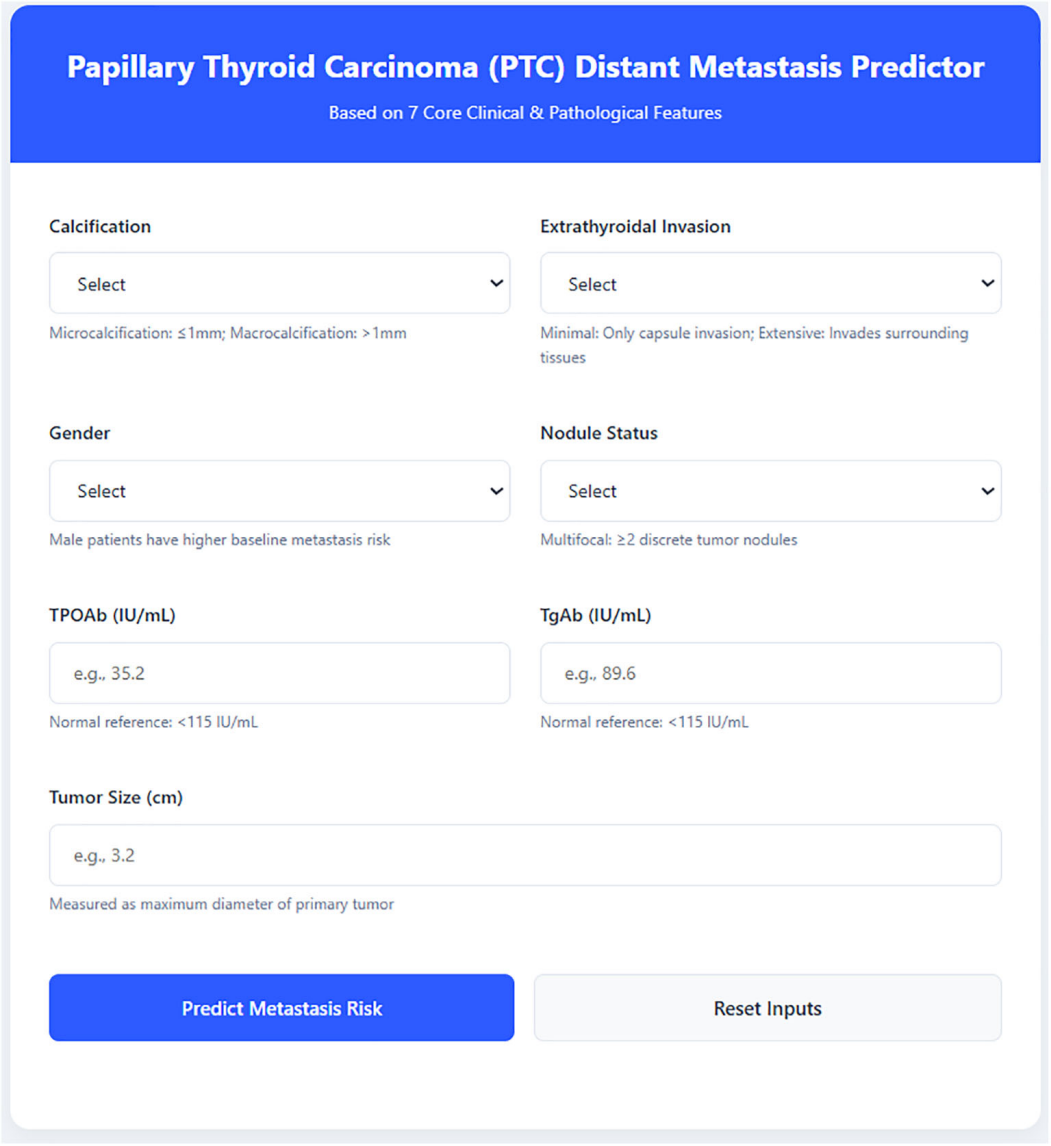
Django-based prediction website.

Medical records from 421 thyroid cancer patients were collected from Jimo District People’s Hospital, Qingdao City to validate model stability. The external validation achieved an AUC of 0.755(code and roc curve is in supplementary data), showing a slight decrease in performance but still maintaining clinical value. Based on the LightGBM model, which demonstrated the best predictive performance, a prediction website was developed (as shown below in **Figure 5**) that can rapidly calculate metastasis probability based on the core predictive features, assisting clinical diagnosis.

## Discussion

### Summary of the present study

The present study successfully developed a prediction model for distant metastasis in PTC based on comprehensive clinical data, laboratory indices, and ultrasound imaging features. Through comparison of nine machine learning algorithms, LightGBM was identified as the optimal algorithm, and SHAP analysis provided interpretable feature importance rankings. The model achieved an AUC of 0.886 and a negative predictive value (NPV) of 93.6% on the validation set, indicating excellent potential for clinical application. Robinson and colleagues further revealed the nonlinear relationship between tumor size and metastasis in their research, elucidating the complexity of prediction models (23).

### Comparison with and advantages over other studies

The present study enrolled a large-scale patient cohort, enhancing statistical power and result generalizability compared with single-center studies, and more closely reflects the laboratory indices commonly employed in domestic clinical practice, incorporating more China-specific factors relevant to this population. Compared with the research by Huang and colleagues (7) (2025) based on the SEER database, although their XGBoost model achieved a high AUC of 0.988, this may reflect overfitting or data quality-specific issues, whereas the present study’s LGBM model achieved a validation set AUC of 0.886, which, although slightly lower in absolute value, demonstrates greater clinical robustness. Moreover, due to its superior interpretability, lower computational cost, and robustness to outliers, it is more suitable for clinical application in the domestic setting.

Regarding feature selection methodology, the present study employed the intersection of three methods (LASSO, RFE, and ReliefF) to select core features, proving more robust than traditional single feature selection methods and ensuring that selected features demonstrate high importance across multiple independent evaluation frameworks, reducing false discovery risk from single-algorithm bias, enhancing model reproducibility across different datasets, and mitigating feature selection data leakage risk. With respect to indicator system construction, compared with studies relying solely on imaging features, the present study comprehensively incorporated critical immunological indices such as TSH, TgAb, and TPOAb. According to SHAP analysis results, extrathyroidal invasion served as the strongest prediction indicator (Mean |SHAP| = 0.132911), with its clinical significance thoroughly confirmed in multiple studies. Following extrathyroidal invasion, thyroglobulin antibody (TgAb) ranked second in importance (Mean |SHAP| = 0.098070), with elevated levels positively correlated with distant metastasis risk. Notably, PTC patients with concomitant Hashimoto thyroiditis demonstrated more pronounced TgAb elevation, suggesting enhanced autoimmune response and more prominent immune suppression in the tumor microenvironment, higher circulating tumor cell burden, and consequently increased metastasis risk and worse prognosis. These findings align with the interpretation that elevated TgAb reflects tumor-induced immune tolerance and tumor microenvironment immunosuppression. Multiple prospective studies have confirmed that “elevated TgAb is an important prognostic marker for PTC recurrence and metastasis,” findings further supported by the present results and echoing the conclusions of Gu and colleagues (9), which revealed “immune-inflammatory markers as independent risk factors for lymph node metastasis in PTC,” collectively emphasizing the central role of immune regulation in PTC metastasis (9) (24).

Calcification, nodule status, sex, TPOAb, and tumor size, serving as other core predictive features, also carry clear clinical value: calcification and nodule status represent classic ultrasound indicators for assessing PTC malignancy risk, consistent with conclusions from Lu and colleagues (2023). The SHAP value for tumor size demonstrated a negative association, suggesting a nonlinear rather than simple positive correlation with metastasis risk, potentially due to high-invasiveness subclones in microcarcinomas, more complete surgical resection in larger tumors, or regulation by other factors—a nonlinear relationship providing new perspectives for clinical risk stratification. Additionally, immune-related prognostic biomarkers identified by Chen and colleagues (24), the PTC prognostic nomogram constructed by Cui and colleagues (25), and the XGBoost-based DTC recurrence prediction model developed by Shrestha and colleagues (3) have from different perspectives confirmed the critical value of “clinical-imaging-immunological features” in PTC prognosis assessment, providing indirect support for the rationality of feature selection in the present study.

Regarding model interpretability, the present study integrated SHAP analysis to elucidate model decision logic, enabling clinicians to understand the specific contributions of each feature to metastasis risk, enhancing model clinical credibility and acceptance, and achieving transformation from “black box to transparent box.” This approach aligns with the methodology employed by Redlich and colleagues (20) (2025) in applying XGBoost + SHAP methods for DTC recurrence prediction in children, confirming the broad applicability of interpretability analysis across different age groups of PTC patients. Meanwhile, the present study also attended to cutting-edge directions in deep learning and multimodal fusion; although deep learning was not directly employed, the high AUC values achieved by Li and colleagues (26) (2025) utilizing artificial intelligence models based on gene expression and by Zhang and colleagues (17) (2022) employing deep learning on dynamic ultrasound videos provided reference directions for subsequent multimodal fusion research. Regarding clinical utility, the present study’s model was constructed based on routine clinical examination and serological indices, requiring no complex genetic testing; compared with classifiers based on mRNA expression by Golding and colleagues (27) (2025) (which, while achieving higher NPV, carry high detection costs and present challenges for grassroots implementation), the present model demonstrates greater clinical accessibility. Furthermore, with validation set specificity of 0.925 and NPV of 93.6%, the model can accurately exclude low-risk patients, substantially reducing overtreatment and related complications, bearing important practical value for enhancing patient quality of life. Additionally, the key features identified in the present study align highly with existing clinical guidelines—extrathyroidal invasion as a core predictive factor corresponds with high-risk factors in the American Thyroid Association (ATA) guidelines and China’s thyroid cancer management guidelines, and aligns with important predictive factors identified by Lu and colleagues (28) (2023), including “thyroid capsule invasion” and “lymph node microcalcifications,” further validating the clinical relevance of the model.

### Clinical and biological significance of key associated features

Through SHAP analysis, the present study clarified the importance and mechanisms of action of core features. Extrathyroidal invasion served as the strongest predictive indicator (Mean |SHAP| = 0.132911), with its clinical significance thoroughly confirmed in multiple studies; patients with capsular invasion demonstrate significantly increased distant metastasis risk, reflecting not only enhanced local tumor infiltration capacity but also suggesting vascular invasion, lymphatic invasion, and other high-metastasis tendencies, marking significant malignant change in tumor biological behavior. Following extrathyroidal invasion, elevated thyroglobulin antibody (TgAb) ranked second in importance (Mean |SHAP| = 0.098070), demonstrating positive correlation with distant metastasis risk. Particularly in PTC patients with concomitant Hashimoto thyroiditis, TgAb elevation is more pronounced, suggesting stronger autoimmune response and consequently more prominent immune suppression in the tumor microenvironment, higher circulating tumor cell burden, increased metastasis risk, and worse prognosis. This phenomenon aligns with the interpretation that elevated TgAb reflects tumor-induced immune tolerance and tumor microenvironment immunosuppression. Multiple prospective studies have confirmed that “elevated TgAb serves as an important prognostic marker for PTC recurrence and metastasis,” conclusions further supported by the present findings and echoing the conclusions of Gu and colleagues (9) (2024), which revealed “immune-inflammatory markers as independent risk factors for lymph node metastasis in PTC,” collectively underscoring the central role of immune regulation in PTC metastasis (9, 24).

Calcification, nodule status, sex, TPOAb, and tumor size, as other core predictive features, also possess clear clinical value: calcification and nodule status are classic ultrasound indicators for assessing PTC malignancy risk, consistent with findings from Lu and colleagues (2023). The SHAP results for tumor size suggest a nonlinear rather than simple positive correlation with metastasis risk, indicating that the association between size and risk may be more complex than traditionally assumed. While possible explanations could involve biological heterogeneity in microcarcinomas, differences in surgical management of larger tumors, or modulation by other clinical and immunological factors, these hypotheses remain speculative; thus, the observed nonlinear pattern should be regarded as hypothesis-generating and interpreted with caution, and further studies are needed to determine whether it can meaningfully refine clinical risk stratificationFurthermore, immune-related prognostic biomarkers identified by Chen and colleagues (24), the PTC prognostic nomogram developed by Cui and colleagues (25), and the XGBoost-based DTC recurrence prediction model by Shrestha and colleagues (3) have from different angles confirmed the critical value of “clinical-imaging-immunological features” in PTC prognosis assessment, providing indirect support for the rationality of feature selection in the present investigation.

### Limitations and future directions

Although the present study achieved significant results, substantial limitations remain. For example, limited representativeness of data sources necessitates multicenter validation to ensure model generalizability; some patients had relatively short follow-up periods with incomplete observation of long-term metastasis; molecular pathological markers (such as BRAF and RET/PTC fusion genes) were not incorporated.

Future research should integrate deep learning multimodal analysis, multi-omics data fusion, multicenter prospective validation, and molecular pathological markers to further enhance prediction accuracy and clinical translation value of the model. Evans and colleagues demonstrated new directions in combining dynamic radiomics and deep learning (29). Metabolism-based prediction models by Du and colleagues (30) (2023) provided new perspectives on metabolism-related gene signatures, and artificial intelligence–driven molecular subtype classification demonstrated by Zhang and colleagues (31) (2025) showcased broad application potential for these emerging technologies in providing more precise biological basis and clinical decision support for PTC metastasis prediction (29–31). Furthermore, the potential of circulating biomarkers in early detection of PTC distant metastasis demonstrated by Brooks and colleagues (32, 33) (2025) merits in-depth exploration in subsequent investigations.

## Data Availability

The full dataset can be provided for researchers if needed by the corresponding author. Requests to access these datasets should be directed to qlyylxg2025@163.com.
